# Supplementary figures and images for: Glycolytic preconditioning in astrocytes mitigates trauma-induced neurodegeneration
Source: eLife. 2021 Sep 2;10:e69438. doi: 10.7554/eLife.69438 (PMC8448530; doi:10.7554/eLife.69438)

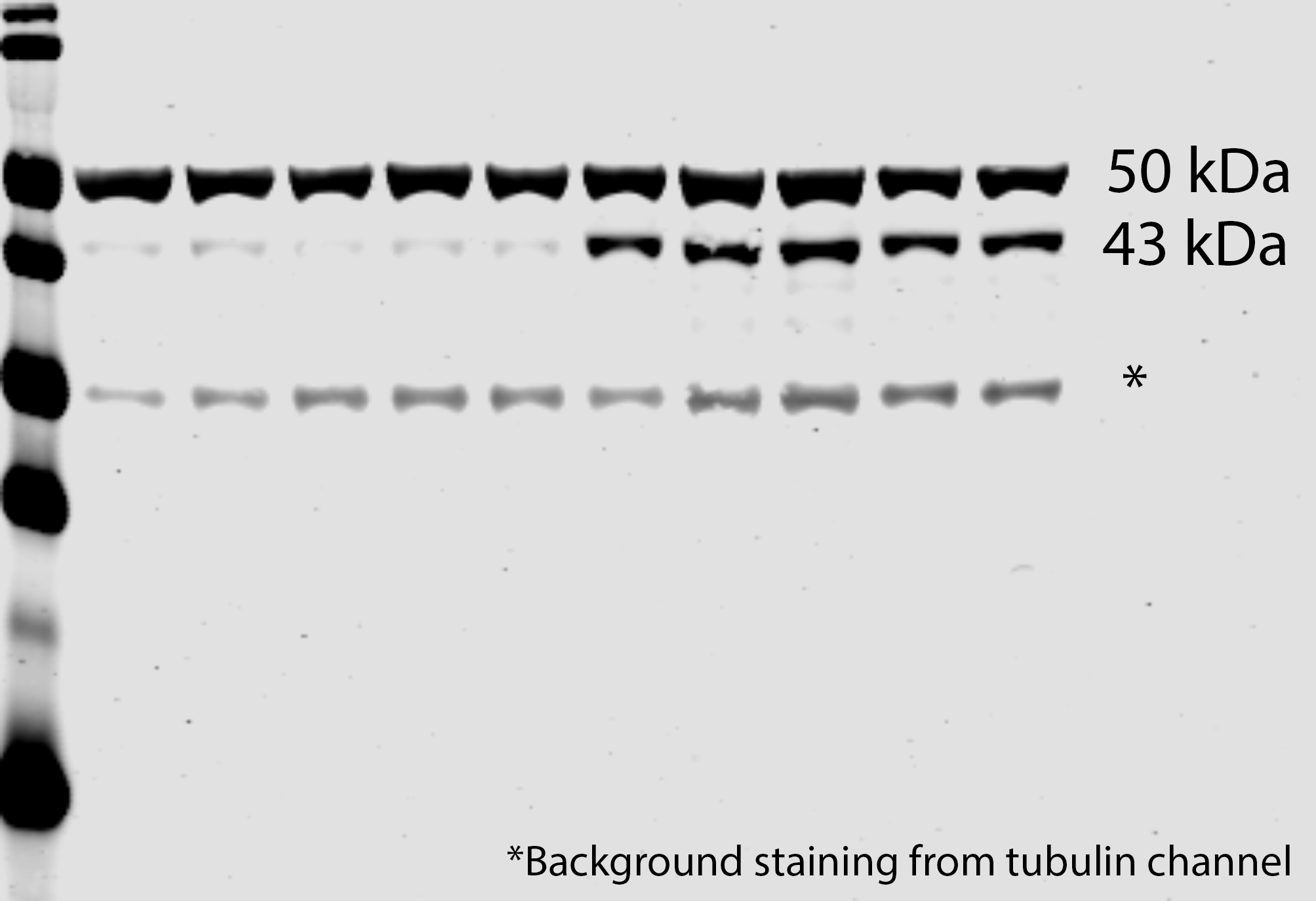

Supplement: Source data 1. [file elife-69438-data1.zip › Figure 5B-source data 1.tif]

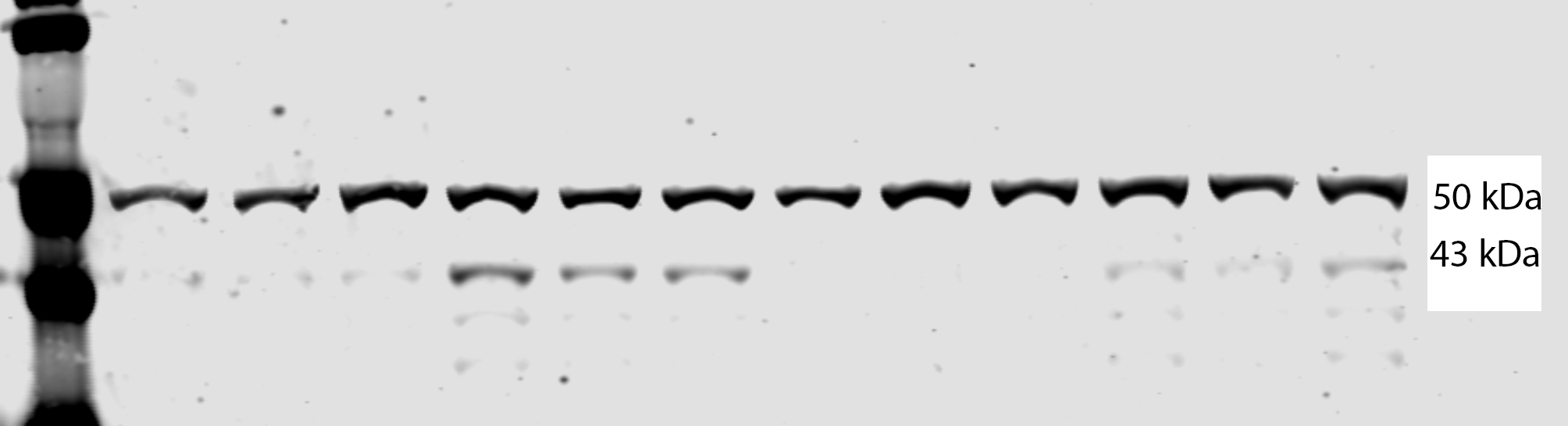

Supplement: Source data 1. [file elife-69438-data1.zip › Figure 5-figure supplement 2A-source data 1.tif]

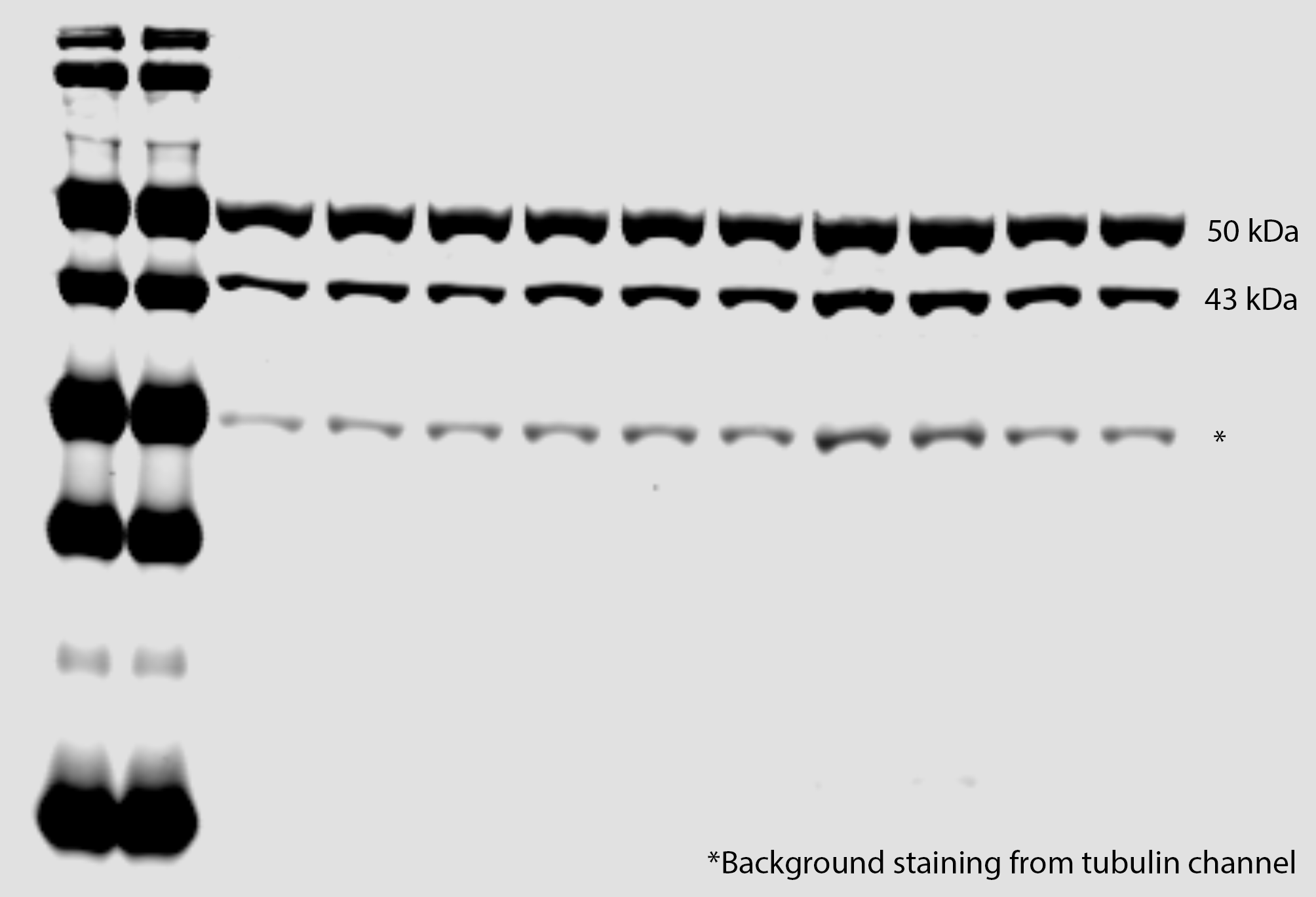

Supplement: Source data 1. [file elife-69438-data1.zip › Figure 5-figure supplement 2B-source data 2.tif]

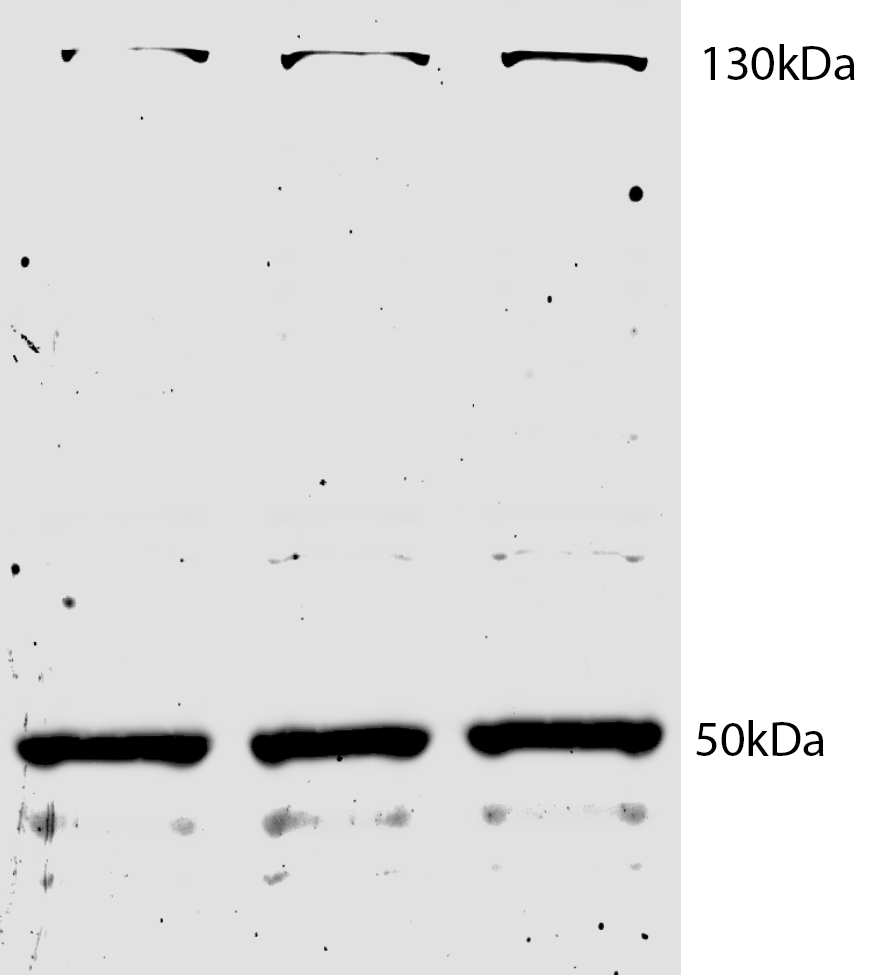

Supplement: Source data 1. [file elife-69438-data1.zip › Figure 5-figure supplement 5J-source data 1.tif]

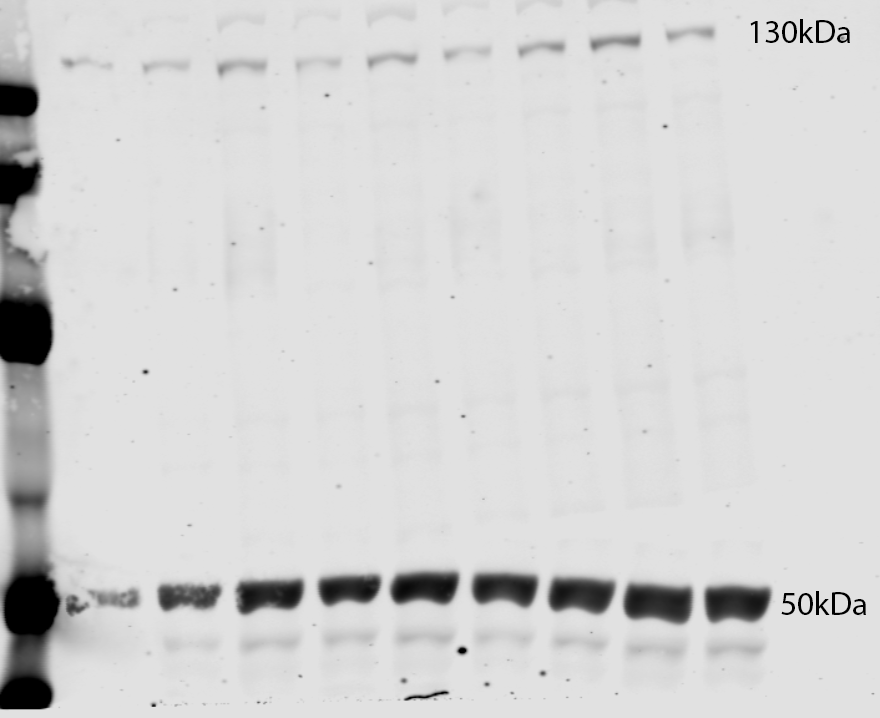

Supplement: Source data 1. [file elife-69438-data1.zip › Figure 5-figure supplement 5K-source data 2.tif]

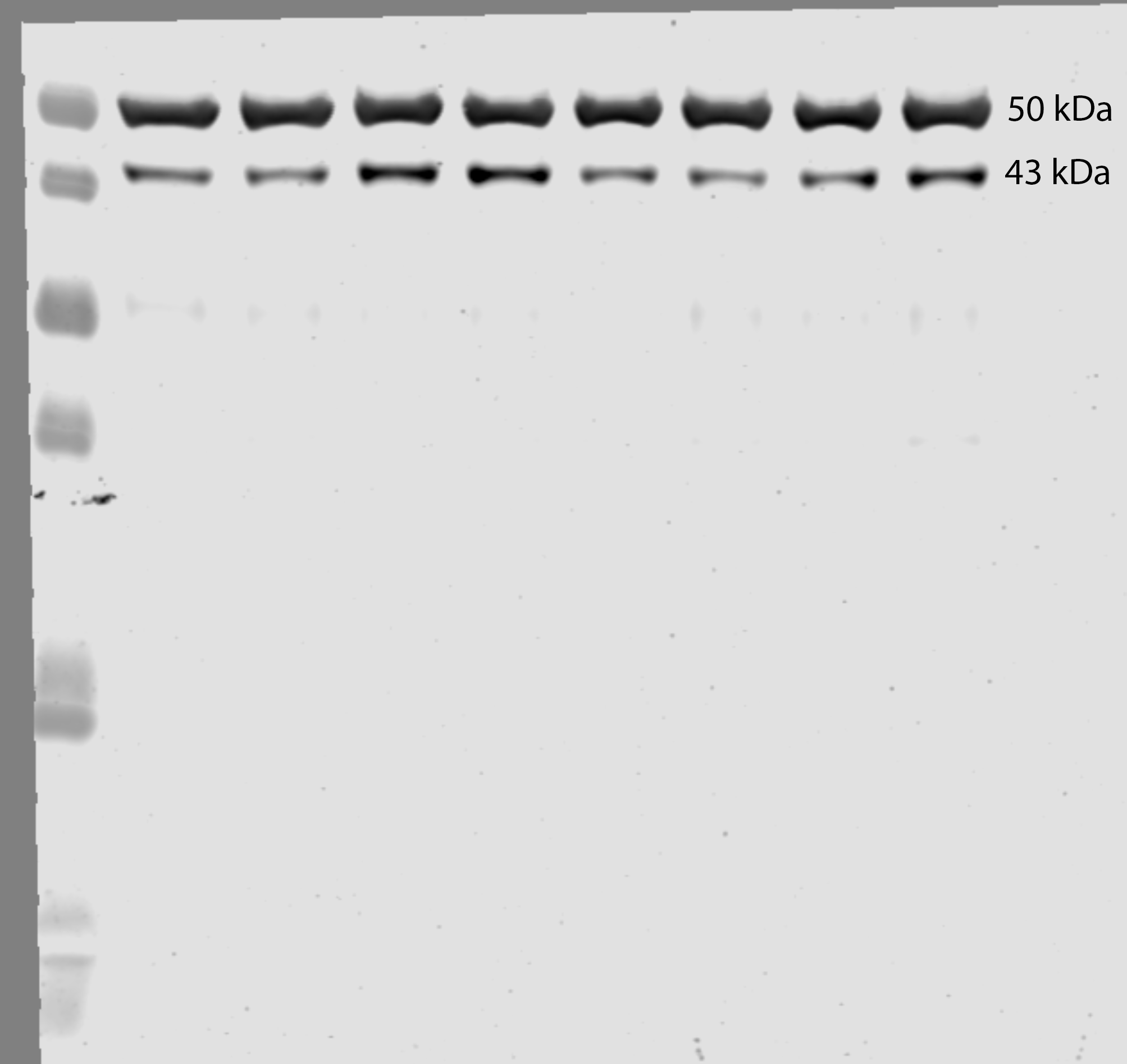

Supplement: Source data 1. [file elife-69438-data1.zip › Figure 5-figure supplement 5R-source data 3.tif]
